# When the Heart, Kidneys, and Body Waste Away: A Review of Cachexia in Cardiorenal Syndrome

**DOI:** 10.1007/s11897-025-00711-2

**Published:** 2025-08-28

**Authors:** Kunaal S. Sarnaik, Saeid Mirzai

**Affiliations:** 1 Department of Medicine, Johns Hopkins University School of Medicine, Baltimore, MD USA; 2 Department of Cardiovascular Medicine, Medical Center Blvd, Wake Forest University School of Medicine, Winston-Salem, NC 27157 USA

**Keywords:** Cachexia, Cardiorenal syndrome, Wasting continuum, Malnutrition, Sarcopenia, Frailty

## Abstract

**Purpose of Review:**

Cardiorenal syndrome refers to disorders of the heart and kidneys in which dysfunction in one organ has resulted in dysfunction of the other. Wasting continuum disorders such as cachexia are highly prevalent in cardiorenal syndrome, yet the clinical impact, pathophysiological mechanisms, and management options have not been well elucidated in previous literature. In this review, we aim to summarize current knowledge regarding the epidemiology, clinical impact, and pathophysiology of CRS-induced wasting continuum disorders, as well as highlight effective and potentially emerging treatment options.

**Recent Findings:**

Neurohormonal activation, inflammation, metabolic dysfunction, gastrointestinal abnormalities, protein degradation, and mitochondrial pathway dysfunction are pathophysiologic mechanisms underlying CRS-induced cachexia. Recent studies have investigated various treatment options targeting such mechanisms with mixed results.

**Summary:**

Early screening of wasting continuum disorders in CRS, in combination with nutritional supplementation and exercise rehabilitation strategies, is the mainstay of management. Pharmacologic optimization may also benefit patients. Future studies are necessary to improve generalizability and consensus definitions of cardio- and renal-specific wasting continuum disorders.

## Introduction

The prevalence of chronic diseases such as heart failure (HF) and chronic kidney disease (CKD) has risen over recent years due to improved life expectancy rates and treatment efficacy [1, 2]. These trends have drastic consequences for not only mortality, but also quality of life and healthcare costs. The heart and kidneys are highly interdependent, and diseases such as HF and CKD share common pathophysiological pathways. Patients who experience dysfunction in one such organ system are thus at a higher risk of developing adverse outcomes in the other organ system [3]. Cardiorenal syndrome (CRS) is an umbrella term utilized to describe the bidirectional and simultaneous dysfunction of the heart and kidneys. CRS encompasses disorders in which acute or chronic dysfunction in either the heart or kidneys induces acute or chronic dysfunction in the other [3]. Due to the prevalence trends of HF and CKD, the consequent rise in the incidence and prevalence of CRS is of urgent concern [4].

Cachexia is a disorder that shares mechanisms with both chronic HF (CHF) and CKD [5]. Broadly, cachexia refers to the wasting of body tissue, typically both lean body and adipose tissue, due to a systemic imbalance between anabolism and catabolism and associated with an underlying chronic disease [6]. Cachexia has been demonstrated to be highly prevalent in several chronic diseases, including cancer, chronic obstructive pulmonary disease (COPD), CHF, and CKD [6]. The presence of cachexia in chronic diseases also portends worse outcomes, yet cachexia remains critically underdiagnosed in such conditions [6]. Cardiac cachexia and renal cachexia refer to cachexia induced by chronic dysfunction of the heart and kidneys, respectively. Pathophysiological mechanisms implicated in cardiac and renal cachexia include dysregulated immune and neurohormonal responses, metabolic and mitochondrial dysfunction, gastrointestinal abnormalities, mitochondrial dysfunction, and perturbations of the protein degradation pathway [5, 7, 8].

Healthcare providers will likely encounter an increasing number of patients with CRS complicated by cachexia, termed cardiorenal cachexia syndrome (CRCS), in the coming decades. A better understanding of current knowledge regarding the complex mechanisms underlying the development of CRS and CRCS may improve detection, management, and treatment options. In the present review, we discuss the development of CRS and explore wasting continuum disorders, such as cachexia, that occur in patients with CRS. We examine the clinical impacts and underlying pathophysiological mechanisms of these conditions, as well as strategies for their prevention and treatment. A narrative search of the MEDLINE database was conducted using the following key relevant search words: *(cardiorenal OR (heart failure AND chronic kidney disease)) AND (cachexia OR frailty OR sarcopenia OR malnutrition)*. Studies were selected based on their relevance and potential to inform the research context.

## Cardiorenal Syndrome: Classification and Pathophysiology

The widely accepted description of CRS, defined by the 2010 Acute Dialysis Quality Initiative (ADQI) consensus conference, encompasses disorders of the heart and kidneys in which acute or chronic dysfunction in one organ induces acute or chronic dysfunction in the other [9, 10]. This definition also stratifies CRS into five types.

The first two types of CRS include disorders in which cardiac dysfunction results in renal dysfunction. In *CRS type 1*, also known as acute CRS, an HF syndrome leads to an acute kidney injury (AKI). Examples of HF syndromes include acute decompensated HF (ADHF), acute coronary syndrome (ACS) and arrhythmias complicated by cardiogenic shock, cardiac surgery, or transient valve failures [3, 11]. Estimates of *CRS type 1* prevalence range from 11 to 50% of patients with ADHF [11]. In *CRS type 2*, also known as chronic CRS, chronic cardiac dysfunction leads to CKD. Examples of inciting cardiac disorders of *CRS type 2* include CHF with reduced or preserved ejection fraction, ischemic cardiomyopathies, and congenital heart disease [12]. Estimates of *CRS type 2* prevalence suggest that approximately one quarter of patients with CHF develop CKD [13–15]. Several mechanisms have been described in the pathogenesis of *CRS types 1* and *2*. Hemodynamically, the combination of decreased cardiac output from the left heart and increased preload of the right heart begins a cascade of events involving the sympathetic nervous system (SNS) and the renin-angiotensin-aldosterone system (RAAS) activation [3, 16]. Furthermore, cytokine secretion from humoral signaling and ineffective actions of natriuretic peptide secretion decrease sodium and water excretion from the kidneys [3, 16]. Combined, these changes result in prerenal vasoconstriction and increased renal venous pressure, both of which decrease renal perfusion pressure and incite damage to the kidneys (e.g., AKI) [3, 16]. Such repeated insults over time may then result in parenchymal damage, followed by sclerosis and fibrosis of the kidneys and the onset of CKD [16].

The next two types of CRS involve disorders in which dysfunction of the kidneys results in dysfunction of the heart. These two types of CRS are thus known collectively as renocardiac syndromes. In *CRS type 3*, also known as acute renocardiac syndrome, AKI leads to acute cardiac dysfunction such as HF, arrhythmias, or ischemia [9]. Estimates of *CRS type 3* prevalence suggest up to approximately 55% of patients with AKI have associated acute cardiovascular sequelae such as HF [11]. Mechanisms underlying *CRS type 3* include volume overload leading to congestion, systolic dysfunction due to damage from uremia, acidosis, or inflammation, and arrhythmogenic effects of electrolyte derangements associated with AKI, such as hyperkalemia [5, 17]. In *CRS type 4*, also known as chronic renocardiac syndrome, CKD leads to chronic cardiac dysfunction due to factors including deleterious cardiac remodeling, arrhythmias, and accelerated atherosclerosis leading to an increased risk of adverse cardiovascular events [3, 5, 9, 11, 17]. A large-scale Medicare study demonstrated 2-year incidence rates for atherosclerotic vascular disease and CHF in patients with CKD to range from approximately 30–53 per 100 patient-years [18]. Similarly, the prevalence of CHF has been found to increase in a stepwise manner as renal function deteriorates in CKD, with estimates of 65–70% prevalence in end-stage renal disease (ESRD) [19]. Pathophysiological mechanisms implicated in *CRS type 4* include those for *CRS type 3*, as well as anemia, calcium and phosphate derangements, oxidative stress, and endothelial dysfunction [20]. Furthermore, as CKD progresses to the dialysis-dependent stage, the hemodynamic stress of dialysis likely contributes to the pathogenesis of *CRS type 4* [20]. Uremic toxins like indoxyl sulfate may also accelerate functional decline in coexisting CHF and CKD [21].

In *CRS type 5*, also known as secondary CRS, acute or chronic systemic disorders inflict simultaneous damage on the heart and kidneys, resulting in dysfunction. Examples of such disorders include sepsis, amyloidosis, diabetes, systemic lupus erythematosus, and sarcoidosis [3, 9]. Out of all CRS types, epidemiology data regarding *CRS type 5* is scarcest, likely due to the systemic multi-organ failure beyond just that of the heart and kidneys that may manifest. Furthermore, the pathogenesis of *CRS type 5* is dependent on the inciting disorder; given the myriad of such disorders and the scope of the current review, only sepsis is discussed here. Sepsis inflicts renal dysfunction secondary to the inflammatory response and vasodilation, resulting in decreased renal perfusion pressure and kidney damage [22]. Cardiac dysfunction also manifests due to direct myocardial toxicity of the inflammatory response, as well as tachyarrhythmias [22]. The aforementioned cross-talk between the kidney and heart, discussed in *CRS types 1–4*, ensues and compounds the damage [22].

### The Wasting Continuum

When discussing cachexia, it is important to distinguish between related disorders along the wasting continuum. Malnutrition, myopenia, dynapenia, sarcopenia, frailty, and cachexia are somewhat clinically, prognostically, and mechanically distinct, yet they may present similarly (Table 1) [23]. The wasting continuum hypothesis posits that skeletal muscle is lost before adipose tissue [24]. Thus, according to this hypothesis, the sequence of wasting in chronic diseases typically starts with loss of muscle quantity (myopenia) and/or strength (dynapenia), both of which combined result in sarcopenia. Wasting may then progress to generalized catabolism and cachexia, with the risk of mortality successively increasing [24]. In CKD, protein-energy wasting (PEW), a condition in which both marked catabolism and undernourishment coexist, has also been described [25]. Marked by more moderate changes in serum chemistry, body mass, muscle mass, and dietary intake relative to that of cachexia, as outlined by the International Society of Renal Nutrition and Metabolism (ISRNM) [25], PEW may precede cachexia [26]. Frailty and malnutrition, on the other hand, may develop at any period along this sequence.

**Table 1:** Overview of wasting continuum disorders with their descriptions, diagnostic criteria, common features, and distinguishing characteristics

Wasting Disorder	Description/Definition	Diagnostic Criteria	Common Features	Distinguishing Characteristics	Additional Comments
Malnutrition	- Imbalances in the intake of energy or nutrients, potentially resulting in excess or deficiencies of nutrients [27]	− 2019 Global Leadership Initiative on Malnutrition (GLIM) [28] - Phenotypic criteria include [28]: 1) non-volitional weight loss 2) low body mass index 3) reduced muscle mass 4) reduced food intake or assimilation 5) burden of disease or inflammatory conditions	- Muscle and strength loss [23], [29] - May not necessarily have loss of fat [6], [23]	- Typically reversible with nutritional intervention [6] - Inflammation, loss of appetite, and low food intake typically present [23] - More likely if fat loss present [6], [23]	− 2-step procedure for screening and diagnosis [28] - Stratifies malnutrition based on severity of phenotypic criteria into Stage 1 (moderate) malnutrition and Stage 2 (severe) malnutrition [28] - Extensively validated in various cohorts [30]
Myopenia	- Low muscle mass - Disease-specific and clinically relevant muscle wasting associated with poor functional status, morbidity, and mortality [31]	- No consensus-derived set of diagnostic criteria - Individual investigations define myopenia as either [31], [32]: 1) degree of muscle loss over time 2) muscle mass below prespecified threshold		- Weight loss and low BMI not necessarily present [23]	
Dynapenia	- Loss of muscle strength that cannot be explained by neurologic or muscular diseases [33]	- No consensus-derived set of diagnostic criteria - Suggested by presence of poor test results on [34]: 1) hand grip strength 2) timed get up and go 3) short physical performance battery			
Sarcopenia	- Progressive loss of muscle mass and strength secondary to age or underlying disease [35]	- European Working Group on Sarcopenia in Older People (EWGSOP-2) criteria [36] - Sarcopenia present if both of following are present [36]: 1) low muscle strength 2) low muscle quantity or quality - Qualifier of ‘severe’ added if evidence of decreased physical performance is present [36]			- Can be thought of as combination of myopenia and dynapenia - Examples of tests for [36]: 1) low muscle strength: hand grip 2) muscle quantity: lumbar muscle cross-sectional area by computed tomography scan 3) muscle performance: gait speed
Frailty	- Syndrome in which age or disease results in the decline of function and physiologic reserve across multiple systems with increased vulnerability to stressors [23]	- No gold standard diagnostic criteria - Clinical assessment with the Clinical Frailty Scale (CFS) [37], [38]			- CFS stratifies frailty based on individual disease symptoms, activity, ability to carry out activities of daily living, and life expectancy [37], [38]
Cachexia	- Generalized wasting secondary to an imbalance between anabolic and catabolic processes [6] - Associated with underlying disease [6]	- Evans et al. criteria [39] - Cachexia diagnosed if non-edematous weight loss of greater than or equal to 5% within 12 months in the presence of an underlying illness, in addition to 3 of the following 5 criteria [39]: 1) decreased muscle strength 2) fatigue 3) anorexia 4) low fat-free mass index 5) abnormal biochemistry in the form of either: a) increased inflammation (e.g., C-reactive protein > 5.0 mg/L or IL-6 > 4.0 pg/mL) b) anemia (hemoglobin < 12.0 g/dL) c) hypoalbuminemia (albumin < 3.2 g/dL)		- Not amenable to nutritional intervention alone [6] - Inflammation, loss of appetite, and low food intake typically present [23] - More likely if fat loss present [6], [23]	

## Epidemiology and Clinical Impact of Cachexia in CRS

**Fig. 1:**
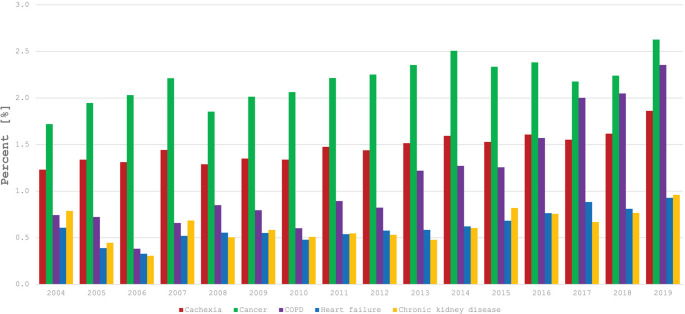
Prevalence of cachexia at discharge or death from 2004 to 2019 in patients with cancer, chronic obstructive pulmonary disease, heart failure, and chronic kidney disease obtained from retrospectively analyzing the National Hospital Health Care Statistics Database. From Lainscak et al. [54] licensed under CC BY. COPD = chronic obstructive pulmonary disease

A substantial proportion of patients with chronic heart and/or kidney dysfunction have wasting disorders [8, 40]. Frailty has been estimated to affect approximately 15–74% of patients with CHF [41], and sarcopenia prevalence has been estimated to be 10–68% [42], the wide ranges likely a result of heterogeneity in the measurement systems utilized [40]. With regard to malnutrition in CHF, a recent meta-analysis of 31 studies calculated an estimated prevalence of 46% [43]. Cachexia is similarly present to a high degree in CHF, with two studies utilizing the diagnostic criteria outlined above by Evans et al. [39] estimating prevalences of 35.5% [44] and 32.7% [45]. In CKD, the global prevalence of malnutrition has been estimated to be 42.7% [46], and the prevalence of PEW has been estimated to be 31% in adults [47]. Similar to that of CHF, prevalence estimates of sarcopenia and frailty in CKD vary, with estimates ranging from 3.9 to 98.5% and 15–21%, respectively [48]. Cachexia has an estimated prevalence of 30–60% in CKD [49]. Accordingly, wasting disorders in CRS are also present to a high degree, though studies are relatively scarcer with smaller samples. An investigation of 22 patients with CRS demonstrated malnutrition to likely be present in 45% and cachexia to be present in approximately 36.3% [50]. In a study of 103 patients with coexisting CKD and CHF, frailty was identified in 49.5% of the cohort [51]. A similar analysis demonstrated that patients with coexisting CHF and CKD had poorer nutritional status with lower serum albumin and more severe anemia relative to patients with CHF but without CKD [52]. Furthermore, findings from Nguyen et al. demonstrated that cardiovascular disease in patients with CKD leads to a higher burden of geriatric syndromes such as frailty and malnutrition [53]. Importantly, wasting syndromes such as cachexia have been demonstrated to be critically underdiagnosed in diseases such as CHF and CKD, and these calculated prevalences are likely underestimates of their true prevalence in CRS (Fig. 1) [54].

Nevertheless, there is a myriad of evidence substantiating the detrimental consequences of wasting disorders in CHF, CKD, and CRS. Malnutrition was demonstrated to worsen left ventricular remodeling in acute HF secondary to potential myocardial infarction in a study wherein 22% of patients also had CKD [55]. Signs of cachexia have also been demonstrated to negatively impact survival in CHF and CKD [56]. In patients with CHF, CKD has also been implicated as a predictor of iron deficiency, the coexistence of which portends worse outcomes, including all-cause mortality and first HF rehospitalization [57]. Frailty was found to portend reduced health-related quality of life in coexisting CKD and CHF [51], and patients with CKD and frailty have been demonstrated to experience worse kidney function, increased mortality, increased cardiovascular disease events, and increased healthcare utilization compared to those with CKD but not frailty [58, 59]. Similarly, in the aforementioned study of patients with CRS, malnutrition status was also associated with higher 6-month mortality rates [50]. Furthermore, a recent investigation demonstrated the presence of CRS and cardiac cachexia to be major criteria (86% and 100% agreement among expert clinicians, respectively) for palliative care referrals in patients with CHF [60].

## Pathophysiological Mechanisms of Cachexia in Cardiorenal Syndrome

Pathophysiological mechanisms implicated in CRCS include neurohormonal activation, inflammatory activation, metabolic dysfunction, gastrointestinal abnormalities, and dysfunction of the protein degradation and mitochondrial pathways.

### A. NeurohormonalMechanisms

**Fig. 2:**
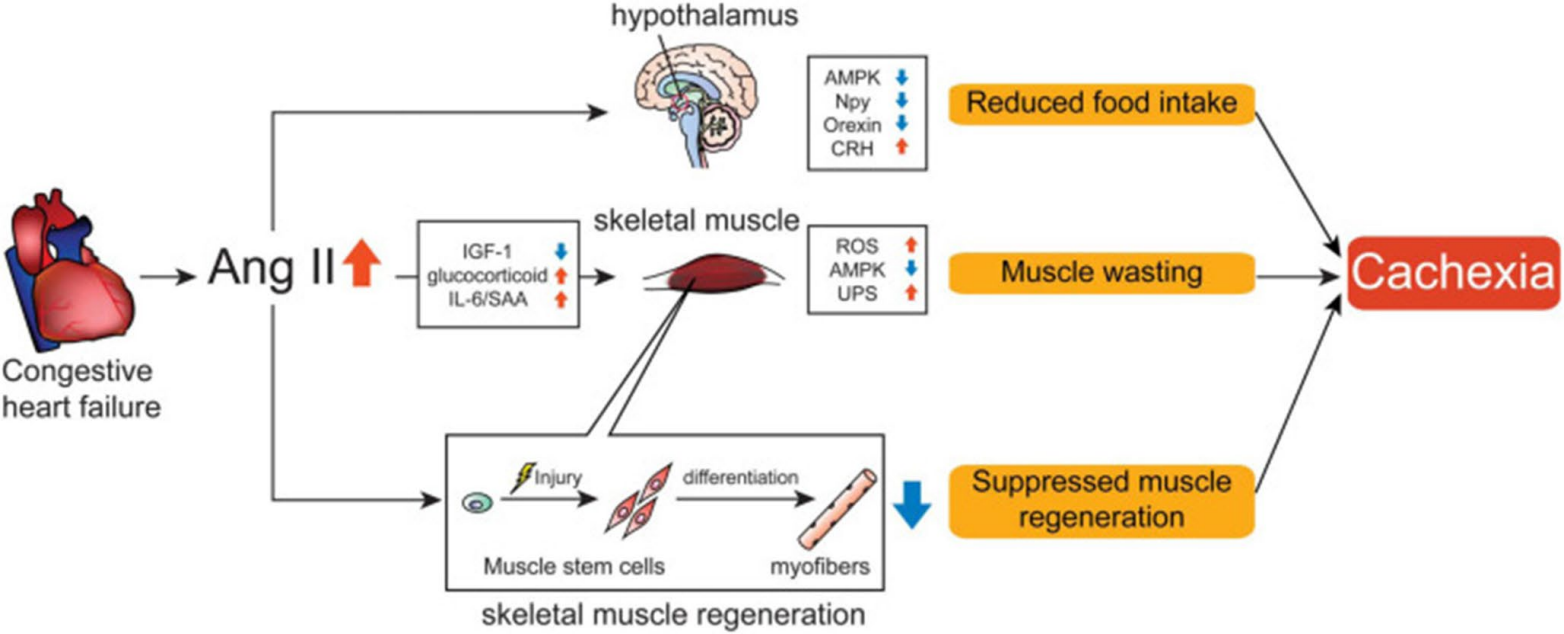
Increased angiotensin II in cardiorenal syndrome plays a key role in the pathogenetic development of cachexia through reduced food intake and altered protein level homeostasis. From Yoshida & Delafontaine [61], with permission; © Elsevier Science & Technology Journals. AMPK = adenosine monophosphate-activated kinase 1; Ang II = angiotensin II; CRH = corticotropin-releasing hormone; IGF-1 = insulin-like growth factor 1; IL-6 = interleukin 6; Npy = neuropeptide Y; ROS = reactive oxygen species; SAA = serum amyloid-A; UPS = ubiquitin-proteasome system

The RAAS and SNS pathways are activated in CRS in response to decreased cardiac output, creating deleterious left ventricular remodeling that perpetuates further neurohormonal activation [5]. RAAS-mediated cachexia occurs through angiotensin II, which reduces food intake and increases skeletal muscle proteolysis (Fig. 2) by perturbing multiple mediators: increasing interleukin (IL)−6, glucocorticoids, corticotropin-releasing hormone, and nicotinamide adenine dinucleotide phosphate (NADPH) oxidase activation while decreasing insulin-like growth factor 1 (IGF-1), orexin, and neuropeptide Y [7, 61]. Angiotensin II also impairs muscular satellite cell activation, disrupting muscular fiber regeneration [7, 62]. Furthermore, SNS hyperactivation overwhelms parasympathetic tone, increasing energy expenditure and catabolism [7]. Beta-2-adrenergic receptor overactivation reduces anabolic stimuli [63], while SNS-mediated peripheral vasoconstriction decreases skeletal muscle blood flow, compounding muscle wasting [63]. The SNS also promotes white adipose tissue wasting [64]. These neurohormonal changes also create cascading growth factor dysfunction through decreased IGF-1 secretion, causing acquired growth hormone (GH) resistance and net protein catabolism [5, 8]. Furthermore, intracellular insulin receptor substrate 1 (IRS1) signaling pathway dysfunction renders cells essentially unresponsive to IGF-1, further impairing muscle fiber regeneration since muscular satellite cells require IGF-1 activation [7, 65]. Additional hormonal imbalances compound these effects, including decreased testosterone, which worsens anabolism and contributes to GH resistance through reduced IGF-1 mRNA expression in muscle [5, 65, 66]. Simultaneously, increased ghrelin levels with ghrelin resistance augment GH resistance and promote anorexia [5, 8, 63, 65, 66]. In CKD, increased leptin due to decreased renal clearance further promotes satiety and anorexia [8].

### B. Inflammatory Mechanisms

The inflammatory response, elevated in both CHF and CKD, represents another important mechanism in CRS-induced cachexia. Pro-inflammatory cytokines such as tumor necrosis factor-alpha (TNF-alpha), IL-1, IL-6, and interferon-gamma (IFN-gamma) activate catabolic processes resulting in muscle wasting [7, 8, 24, 63, 65, 66]. These cytokines, influenced by neurohormonal abnormalities such as ghrelin resistance, activate nuclear factor kappa B (NF-kB) and promote downstream ubiquitin-proteasome system (UPS) activity, leading to increased protein degradation [7, 8, 66]. The inflammatory state also creates a hypermetabolic state, suppresses insulin signaling, and increases glucocorticoid secretion, collectively promoting catabolism over anabolism [8]. Hypoalbuminemia, common to both CHF and CKD, reduces anti-inflammatory cytokines such as IL-10 and transforming growth factor beta (TGF-beta), impairing inflammatory regulation and worsening catabolism [8]. Moreover, increased angiotensin II and decreased testosterone reduce anti-inflammatory cytokines like TGF-beta in cardiac cachexia [8, 66].

### C. Metabolic Abnormalities

Insulin resistance, resulting from inflammation and potentially serving as an inciting factor for diabetic CKD in CRS, contributes to net protein catabolism through decreased phosphoinositide-3-kinase (PI3K) intracellular pathway activity, which activates the UPS to degrade skeletal muscle proteins [8]. Vitamin D deficiency and metabolic acidosis commonly complicate CKD in CRS and further contribute to insulin resistance [8]. Increased energy expenditure from increased sympathetic tone, RAAS activation, and inflammation causes catabolic processes that overwhelm anabolic processes, promoting cachexia development [7]. Myostatin, a negative regulator of muscle mass that is overexpressed in CHF and CKD, represents a major contributor to this imbalance [66]. Myostatin levels rise in cardiomyocytes and skeletal muscles due to prolonged cardiac stress and promote NF-kB signaling to increase UPS activity and protein degradation [63]. Uremia further shifts this balance by increasing myostatin levels while decreasing IGF-1 levels [8].

### D. Gastrointestinal Abnormalities

Recent evidence implicates gastrointestinal abnormalities in CRCS pathogenesis. Specifically, the inflammatory response induces thickening of the alimentary tract walls and increased bacterial growth [7]. Impaired microcirculation of the gastrointestinal tract then develops, promoting collagen deposition and resulting in intestinal epithelial cell dysfunction and malnutrition [7]. Venous congestion in CHF and CKD causes bowel wall edema, further separating the intestinal epithelium from mesenteric capillaries and compounding malabsorption of nutrients (e.g., iron, protein, and fat) and pharmacologic therapeutics [63]. Reduced intestinal blood flow increases intestinal bacterial growth, potentially causing bacterial translocation and worsening inflammation through bacterial lipopolysaccharide, an endotoxin that increases TNF-alpha secretion and amplifies the pathogenetic mechanisms described above [62, 66]. These gastrointestinal abnormalities produce symptoms such as nausea, early satiety, belching, and anorexia, further impairing anabolic processes by reducing nutritional intake [66]. Finally, gut microbiome changes alter vitamin absorption and metabolite production, potentially contributing to CRS development and cardiac cachexia [67].

### E. Protein Degradation Pathways

As discussed earlier, the UPS in CRS is overactivated due to inflammation, angiotensin II, and myostatin. Though this results in an upregulation of protein degradation, other pathways also play a role. These include the calcium-dependent lysosomal autophagy, caspase-dependent apoptosis, and matrix metalloproteinase pathways [63]. Angiotensin II has been implicated in increased activity of the calcium-dependent lysosomal autophagy pathway [7]. In CHF, perturbations in Bax and Bcl-2 levels have also been observed, successively resulting in cytochrome c release, caspase activation, and apoptosis [24]. Importantly, in CKD, these increases in caspase activity resulting in apoptosis are not opposed by increased anabolic compensation, promoting a state of net protein and cell catabolism and cachexia [5].

### F. Mitochondrial Dysfunction

Mitochondrial dysfunction also manifests in CRS, which impairs energy production [63]. In uremia, there may also be increased activity of mitochondrial uncoupling proteins, which results in increased thermogenesis and contributes to wasting [8]. The consequent increase in energy expenditure may contribute to the anorexia observed in CRS-induced cachexia [8]. Oxidative stress can lead to mitochondrial damage in muscular tissue in CRS, as the increase in angiotensin II increases NADPH oxidase activation and promotes the release of reactive oxygen species [7]. Inflammation also contributes to the increased oxidative stress present in CRS [5].

### G. The Vicious Circle: Cardiorenal Cachexia Syndrome

**Fig. 3:**
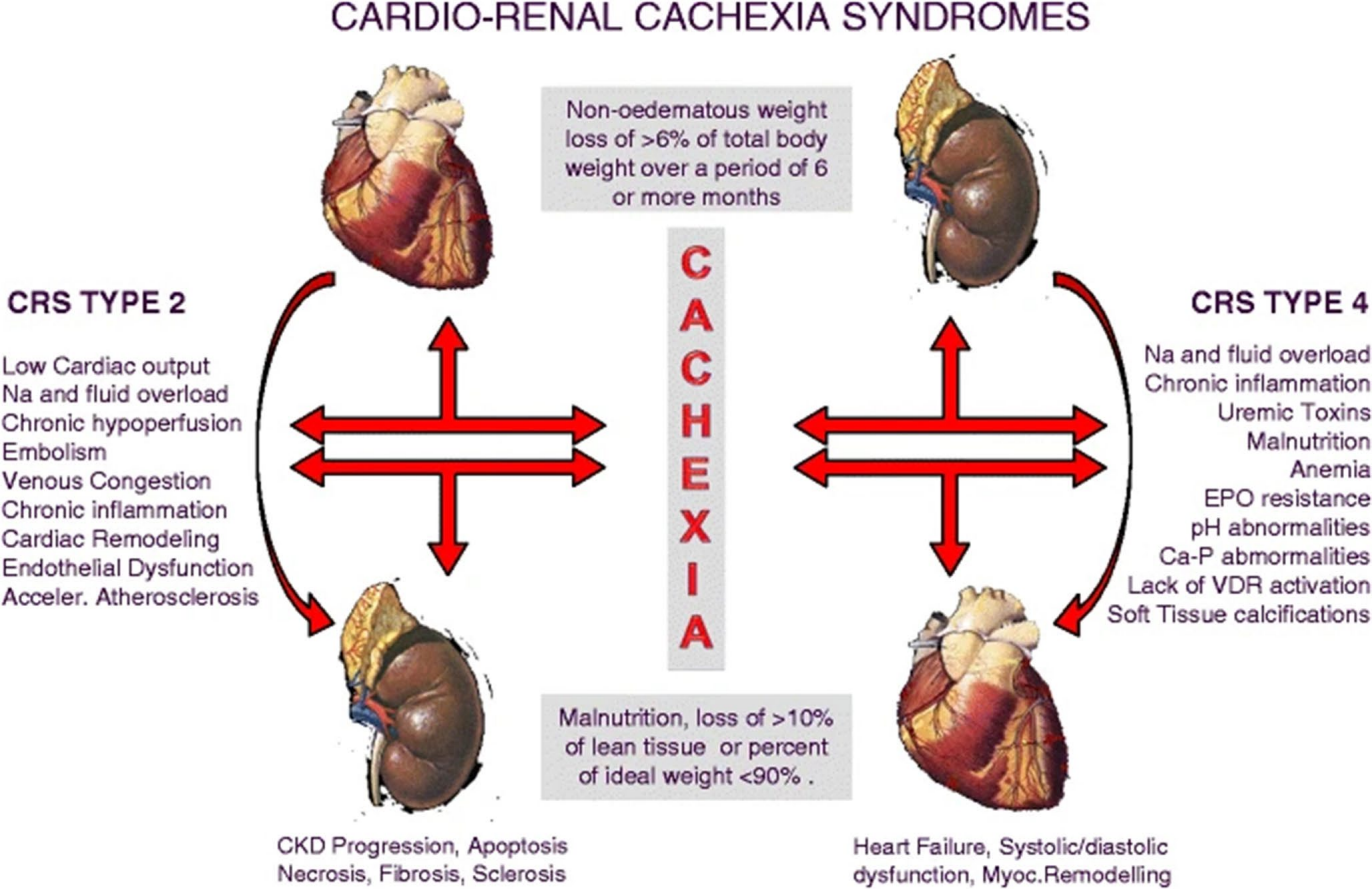
Pathogenetic mechanisms of cardiorenal cachexia syndrome (CRCS). Hemodynamic, neurohormonal, inflammatory, and metabolic abnormalities common to both cardiorenal syndrome (CRS) and cachexia create a devastating cycle that worsens the severity of CRS, as well as that of wasting, with such consequences compounding over time. From Cicoira et al. [5] licensed under CC BY-NC. Ca-P = calcium-phosphate; CKD = chronic kidney disease; CRS = cardiorenal syndrome; EPO = erythropoietin; Na = sodium; VDR = vitamin D receptor

Though the earlier sections focus on how pathogenetic mechanisms of CHF, CKD, and CRS promote cachexia, the reverse relationship also exists (Fig. 3). Specifically, cachexia may result in sustained activation of the immune and neuroendocrine systems, further deteriorating cardiac and renal function via increased peripheral vascular resistance and decreased renal perfusion pressure [5]. As a result, deleterious remodeling of the heart and kidneys may progress more rapidly, further promoting cachexia and manifesting the disastrous cycle of CRCS [5]. Cachexia also alters pharmacokinetic properties, such as decreasing drug absorption, volume of distribution, and albumin levels [66]. Consequently, pharmacologic therapies that serve to combat the pathophysiology of CRS may be rendered ineffective. Additionally, cachexia may lead to decreased cytochrome p450 enzymatic activity and increased drug half-life secondary to hepatic congestion in CRS [66]. This renders patients more susceptible to the adverse effects of pharmacologic therapies, further dampening the potential for recovery of patients with CRS and cachexia in response to drug treatment.

## Prevention and Treatment Strategies

Table 2 summarizes prevention and treatment strategies for CRS-induced cachexia.

**Table 2:** Overview of prevention and treatment strategies for cardiorenal syndrome-induced cachexia

Nonpharmacologic Strategies
Screening for wasting continuum disorders
Nutritional Rehabilitation
Protein supplementation for goal protein intake > 1 g/kg per day (careful with renal function) Essential amino acid, branched-chain amino acid, and amino acid derivative supplementation Omega-3-polyunsaturated fatty acid supplementation Iron repletion Erythropoietin supplementation in anemia Limiting salt and fluid intake Repletion of vitamin D deficiency
Exercise Therapy
Resistance training Aerobic exercise Cardiac rehabilitation
Pharmacologic Strategies
Guideline-directed medical therapy up-titration to maximum tolerable dose
Experimental strategies
Appetite stimulants Ghrelin agonists Anabolic agents (e.g., testosterone, recombinant growth hormone, and selective androgen receptor modulators) Melanocortin signaling pathway antagonists

Given the disastrous pathophysiology and devastating clinical impact of CRCS, early identification of the wasting continuum in patients with CRS is paramount. To achieve this, screening for malnutrition, sarcopenia, cachexia, and frailty must be performed early and often throughout the CRS disease course. Efficient screening strategies validated for malnutrition specifically include the Malnutrition Screening Tool [68], Mini Nutritional Assessment Short Form [69], and Malnutrition Universal Screening Tool [23, 70]. For sarcopenia, the SARC-F questionnaire may be useful, as it quickly assesses for strength, assistance in walking, rise from a chair, climbing of stairs, and fall burden [71]. The CFS may be utilized for frailty screening [37, 38], and the cachexia score (CASCO or miniCASCO) [72, 73] or CASC-IN [74] tools may be utilized for identification of potential cachexia. If such screening assessments yield positive results suggesting the presence of wasting disorders, frameworks described earlier, such as the GLIM model for malnutrition [28] and EWGSOP-2 criteria for sarcopenia [36, 75], should then be utilized for establishing early diagnoses. Useful biomarkers that should be monitored in patients with CRS include CRP, albumin, and hemoglobin levels, as these can efficiently suggest the presence of malnutrition or pre-cachexia [7]. Prealbumin, cholecystokinin, and ghrelin levels may also be useful, yet additional studies are necessary to establish the reliability of these markers [7]. Body composition assessment techniques should also be utilized for assessing the distribution and quantity of muscle and adipose tissue with regard to monitoring for sarcopenia and cachexia, as BMI may not be sufficient to detect the presence of such disorders [23]. Body composition assessment techniques include bioimpedance analysis, cross-sectional segmentation of opportunistic CT or magnetic resonance imaging (MRI) studies, or dual-energy X-ray absorptiometry (DXA) [36]. Additionally, gait speed, hand grip, and chair stand tests may be useful procedures to assess for muscle strength and performance [36] in patients with CRS.

After wasting continuum disorders are identified in patients with CRS, treatment should be readily implemented starting with nutritional rehabilitation. Preliminary trial data regarding dietary protein supplementation in CHF have suggested encouraging results, and expert consensus generally recommends at least 1 g/kg of protein intake per day in patients with CHF [76]. However, clinicians must carefully balance the importance of protein supplementation with that of preserving renal function in patients with CRS [77]. Subgroup analyses of patients affected by kidney dysfunction in ongoing CHF trials such as ASTRID-HF (A Skeletal Muscle Recovery Intervention with Dietary Protein in Heart Failure; NCT05627440), ENOL (Enhanced Nutritional Optimization in LVAD Trial; NCT05655910), and High Calorie High Protein Nutrition Supplementation in Advanced Heart Failure (NCT05219708) may further inform protein intake recommendations [76]. Patients with CRS and evidence of wasting disorders may also benefit from supplementation of essential amino acids, branched-chain amino acids such as leucine, and amino acid derivatives such as beta-hydroxy-beta-methylbutyrate (HMB) [77]. For those patients with decreased adipose tissue along the wasting continuum, omega-3 polyunsaturated fatty acids have also been demonstrated to restore body fat mass [65]. Other nutritional approaches include repleting iron and potentially utilizing erythropoietin in anemic patients with CRS [19], avoiding excessive salt and fluid intake [65], and repleting vitamin D deficiencies [77].

**Fig. 4:**
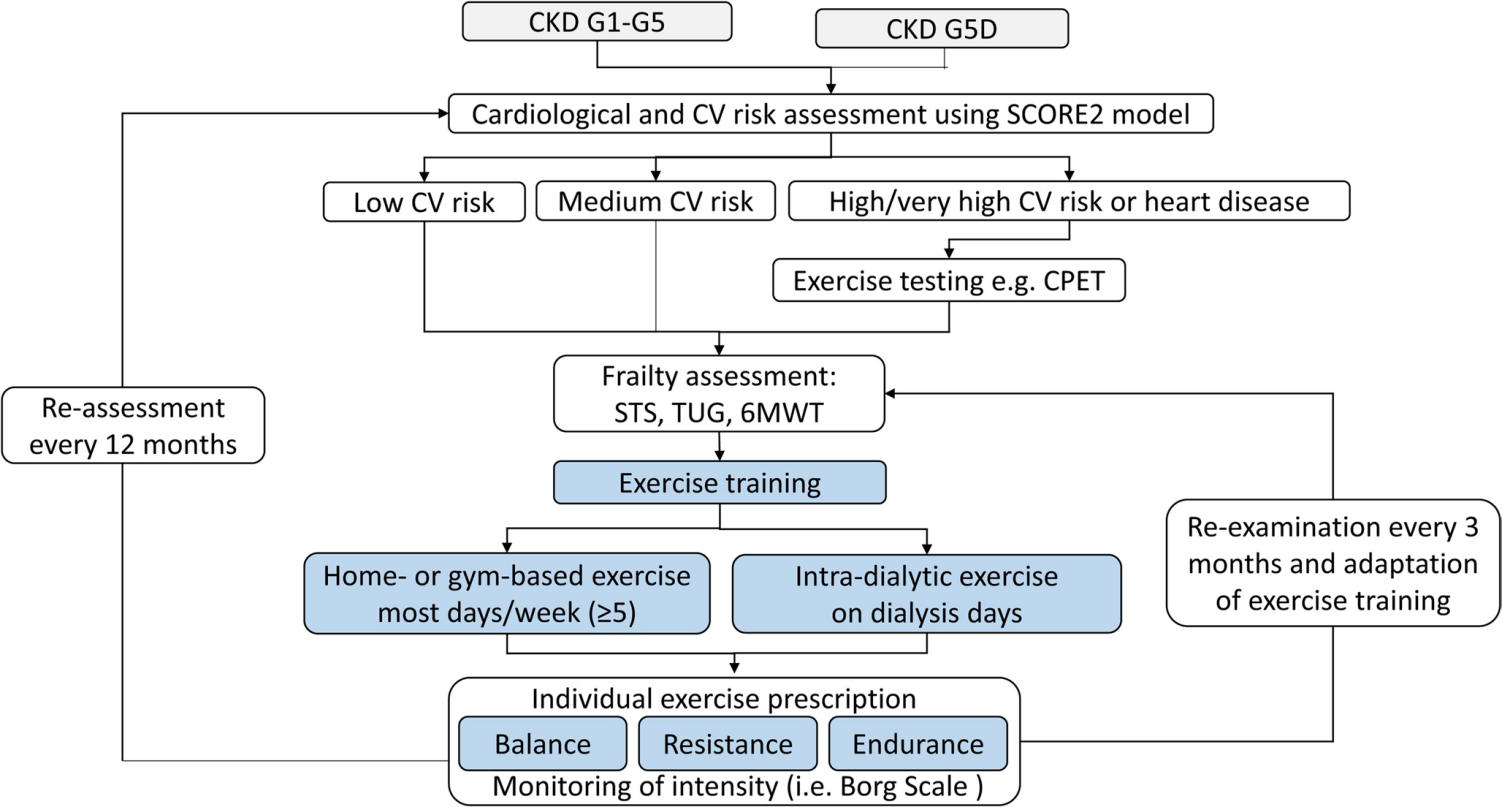
Exercise management algorithm for patients with coexisting heart failure and kidney disease qualified by the presence of frailty. Patients should be stratified based on the severity of chronic disease as well as frailty, and both aerobic and resistance exercise training should be incorporated with periodic re-assessment and re-examination. Exercise testing may also be performed in those patients with higher cardiovascular disease risk or existing heart disease, such that such results may be trended over time and exercise intensity prescriptions can be obtained. From Kouidi et al. [78] licensed under CC BY-NC. 6MWT = six-minute walk test; CKD = cardiovascular disease; CPET = cardiopulmonary exercise testing; CV = cardiovascular; G1-G5 = grade 1 to grade 5; G5D = grade 5 with dialysis; STS = sit-to-stand test; TUG = timed up and go test

Physical exercise interventions are also promising for both the prevention and treatment of muscle wasting disorders, and these should include a combination of aerobic exercise and resistance training in CRS (Fig. 4) [62]. Such training is thought to induce anabolic stimulus that combats the excessive catabolism present in the pathophysiology of CRS-induced wasting [65]. Exercise training has been demonstrated to reduce the cardiovascular disease risk, as well as frailty, in patients with stage 3–5 CKD [78]. Cardiac rehabilitation strategies have also demonstrated efficacy in patients with coexisting HF and CKD to prevent and/or treat wasting disorders, with results of recent studies suggesting improvement in functional status, biomarkers, muscle strength, and muscle performance [79, 80].

Though nutritional and exercise strategies are likely the most evidence-informed management options, emerging pharmacological approaches may also benefit patients with CRS-induced cachexia. First and foremost, guideline-directed medical therapy (GDMT) for both CHF and CKD should be uptitrated to maximum tolerable dosages to combat the inciting pathophysiological mechanisms [65, 81]. Certain GDMTs, such as angiotensin-converting enzyme inhibitors (ACEis) [61] and beta-blockers (BBs) [66], may help combat the neurohormonal activation underlying CRS-induced cachexia. Moreover, ACEis and BBs have demonstrated beneficial effects in partially reversing cardiac cachexia and should thus be prioritized in pharmacologic management [66, 82]. Appetite stimulants such as megestrol acetate have shown benefit with regard to weight gain in cancer cachexia studies, yet results are not as convincing in non-cancer cachexia [66]. For instance, in patients with ESRD, megestrol acetate improved appetite and fat mass at the expense of lean mass [8]. Ghrelin agonists such as anamorelin have also fared well in cancer cachexia studies, yet results have not yet been replicated in cardiac or renal cachexia [65]. Anabolic agents such as testosterone, recombinant GH, and selective androgen receptor modulators, as well as biologics antagonizing TNF-alpha (e.g., etanercept and infliximab) have similarly not been effective in studies of cardiac cachexia [66, 76]. Finally, blockade of leptin and melanocortin signaling via small molecule antagonists has shown promise in alleviating uremia-associated cachexia in mice, yet studies in patients with CKD or CRS have not yet been conducted [8].

Regardless, proper management protocols of wasting continuum disorders in CRS require a multidisciplinary team [23]. Experienced nursing staff form the frontline of this team, as they can effectively screen and identify potential wasting disorders as part of the initial assessment. Nutritionists and physical therapists are vital for proper monitoring and addressing the supplementation and exercise strategies recommended to patients. Pharmacists may also be effective in evaluating the risks and benefits of the various pharmacologic options for each patient. In addition to cardiologists, nephrologists, and geriatricians, the healthcare team should consist of palliative care specialists when necessary, given the high severity of CRS-induced wasting disorders and immense potential for complications. Furthermore, due to the positive association between age and wasting disorder prevalence, patient and caretaker preferences should be accounted for in the management plan.

## Research Gaps and Future Directions

There is a dearth of evidence regarding management options for cardiac and renal cachexia. Many clinical trials in HF have excluded patients with advanced CKD, which has limited the adoption of GDMT in CRS [81]. Compounding this is the relative hesitancy of providers in prescribing GDMT to patients with CRS due to fear of complications such as hyperkalemia, hypotension, and worsening renal function [81]. Similarly, the substantial prevalence of frailty in CRS has also limited GDMT coverage in affected patients, especially since polypharmacy is a well-known risk factor for frailty [81]. All this has led to a lack of knowledge regarding GDMT efficacy for managing not only CRS, but also CRS-induced wasting continuum disorders. Future trials must increase the representation of patients with coexisting CKD and CHF to better understand GDMT efficacy in preventing or treating wasting disorders, as well as establish more optimal guidelines for nutrition supplementation and exercise strategies in this population.

There also exists a lack of consensus among experts regarding the diagnostic criteria of wasting disorders in cardiac and renal disease, specifically [83]. Though the criteria outlined earlier in this review may be utilized to clinically diagnose such disorders, individual studies on wasting in CHF and CKD have utilized a broad heterogeneity of definitions that do not necessarily conform well to one another [40, 83]. Consequently, the reliability of these individual investigation-specific criteria for wasting in cardiac and renal disorders is concerning, and consensus definitions must be established with specific regard to CHF, CKD, and CRS. As understanding and identification of such disorders continues to grow, deployment of machine learning models to establish optimal cutoffs and diagnostic criteria specific to wasting in CRS may be an interesting avenue of future research

## Conclusions

The prevalence of wasting continuum disorders such as malnutrition, sarcopenia, frailty, and cachexia in CRS is concerning. The coexistence of wasting disorders and CRS leads to adverse functional status, quality of life, morbidity, and mortality outcomes. Cachexia and CRS share several common pathophysiological mechanisms, such as neurohormonal activation, inflammation, metabolic dysfunction, gastrointestinal abnormalities, and dysfunction of protein degradation and mitochondrial pathways. These shared mechanisms may result in a devastating pathophysiological cycle of CRCS, wherein CRS worsens wasting and wasting in turn exacerbates the CRS. Early screening and frequent monitoring of malnutrition, frailty, sarcopenia, and cachexia in CRS is paramount to prevent such deterioration and improve clinical outcomes. Nutritional interventions, combination exercise regimens, and GDMT optimization are the mainstays of management of patients with CRS and cachexia. Future trials of cardiac and renal cachexia should increase the representation of patients with CRS, and consensus-derived diagnostic criteria for wasting continuum disorders in cardiac and renal disorders must be established to further inform management.

## Key References

- W. J. Evans et al., “Cachexia: A new definition,” Clinical Nutrition, vol. 27, no. 6, pp. 793–799, Dec. 2008, doi: 10.1016/j.clnu.2008.06.013
  - The paper establishes consensus-derived definitions for identifying and evaluating the severity of cachexia.
- C. M. Prado, J. J. Bell, and M. C. Gonzalez,“Untangling Malnutrition, Physical Dysfunction, Sarcopenia, Frailty and Cachexia in Ageing,” in Interdisciplinary Nutritional Management and Care for Older Adults: An Evidence-Based Practical Guide for Nurses, Ó. G. Geirsdóttir and J. J. Bell, Eds., Cham: Springer International Publishing, 2021, pp. 99–113. doi: 10.1007/978-3-030-63892-4_8
  - The book chapter compares and contrasts disorders along the wasting continuum, such as frailty, malnutrition, cachexia, and sarcopenia
- M. Cicoira, S. D. Anker, and C. Ronco,“Cardio-renal cachexia syndromes (CRCS): pathophysiological foundations of a vicious pathological circle,” Journal of Cachexia, Sarcopenia and Muscle, vol. 2, no. 3, pp. 135–142, 2011, doi: 10.1007/s13539-011-0038-2
  - The paper, to our knowledge, is the only other previously published work discussing the pathophysiology of cardiorenal syndrome-induced cachexia.
- H. Krysztofiak et al., “Cardiac Cachexia: A Well-Known but Challenging Complication of Heart Failure,” CIA, vol. 15, pp. 2041–2051, Nov. 2020, doi: 10.2147/CIA.S273967
  - The paper provides myriad evidence regarding the pathogenetic mechanisms common to both chronic heart failure and cachexia.
- E. Tumelty, I. Chung, S. Hussain, M. A. Ali, H. Addada, and D. Banerjee, “An Updated Review of the Management of Chronic Heart Failure in Patients with Chronic Kidney Disease,” RCM, vol. 25, no. 4, Art. no. 4, Apr. 2024, doi: 10.31083/j.rcm2504144
  - The paper provides an overview of the most recent guideline-based therapy for patients with both chronic heart failure and chronic kidney disease.

## Data Availability

No datasets were generated or analysed during the current study.
